# JCV viruria associates with suboptimal recovery of kidney function three years after living kidney donation

**DOI:** 10.1590/2175-8239-JBN-2021-0148

**Published:** 2022-01-31

**Authors:** Sara Querido, Carolina Ormonde, Teresa Adragão, André Weigert

**Affiliations:** 1 Centro Hospitalar de Lisboa Ocidental, Hospital de Santa Cruz, Unidade de Transplantação Renal, Serviço de Nefrologia, Carnaxide, Portugal.; 2 Hospital do Divino Espírito Santo, Serviço de Nefrologia, Ponta Delgada, Portugal.

**Keywords:** BK Virus, JC Virus, Living Donors, Kidney, Polyomavirus, Vírus BK, Vírus JC, Doadores Vivos, Rim, Polyomavirus

## Abstract

**Introduction:**

Few studies have investigated pre-donation factors that could affect renal recovery after living kidney donation (LKD). We retrospectively investigated the role of John Cunningham virus (JCV) infection and other pre-donation factors on the magnitude of kidney function decline after LKD.

**Methods:**

Urine JCV viral loads, glomerular filtration rate, and blood pressure were evaluated in 60 consecutive LK donors before donation. Suboptimal compensatory hypertrophy was defined as an eGFR <60% of the pre-donation eGFR.

**Results:**

LKD (40% JCV infected) were followed for 3.2±1.6 years. No association was found between age, gender, and baseline hypertension with 1^st^, 2^nd^, 3^rd^, and 4^th^ years post-donation eGFR <60% of the pre-donation eGFR. Mean eGFR recovery at the 3^rd^ year after donation was lower in JCV infected donors vs non-infected donors (61.8% vs 71.0%, p=0.006).

**Conclusion:**

We hypothesized that JCV could shift glomeruli into a hyperfiltration state before nephrectomy, modulating the magnitude of compensatory hypertrophy after donation. Conversely, JCV might curtail the ability of the remaining kidney to promote hyperfiltration. Longer follow up is needed to determine whether JCV viruria ultimately leads to lower eGFR over time or if it is a protective factor for the remaining kidney.

## Resumo

**Introdução:**

Poucos estudos investigaram fatores anteriores à doação que poderiam afetar a recuperação renal após doação renal de doador vivo (LKD, do inglês *Living Kidney Donation*). Investigamos retrospectivamente o papel da infecção pelo vírus John Cunningham (JCV) e outros fatores de risco pré-doação na magnitude do declínio da função renal após LKD.

**Métodos:**

Cargas virais de JCV na urina, taxa de filtração glomerular e pressão arterial foram avaliadas consecutivamente em 60 doadores renais vivos antes da doação. Hipertrofia compensatória subótima foi definida como uma TFGe <60% da TFGe pré-doação.

**Resultados:**

LKD (40% infectados pelo JCV) foram acompanhados por 3,2±1,6 anos. Não foi encontrada nenhuma associação entre idade, sexo e hipertensão basal com a TFGe pós-doação no 1º, 2º, 3º e 4º anos <60% da TFGe pré-doação. A recuperação média da TFGe no 3º ano após a doação foi menor em doadores infectados pelo JCV vs doadores não infectados (61,8% vs 71,0%, p=0,006). **Conclusão**: Levantamos a hipótese de que o JCV pode levar os glomérulos a um estado de hiperfiltração antes da nefrectomia, modulando a magnitude da hipertrofia compensatória após a doação. Por outro lado, o JCV pode limitar a capacidade do rim remanescente de promover a hiperfiltração. É necessário um acompanhamento mais longo para determinar se a virúria por JCV leva, em última instância, a uma menor TFGe ao longo do tempo ou se é um fator de proteção para o rim remanescente.

## Introduction

Living kidney donors have lower estimated glomerular filtration rate (eGFR) since unilateral nephrectomy inevitably leads to reduced renal mass and function^1^. This eGFR reduction has been associated with a rise in blood pressure, increased proteinuria^2,3^, and change in left ventricular mass^4^. Nevertheless, the consequences of eGFR loss after donation remain unclear. Available data regarding long term risks of living kidney donation (LKD) are conflicting and dependent on the selected control group. Some studies reveal a better vital outcome after LKD when compared to matched controls, while other studies show an increased risk of unfavorable outcomes, such as end-stage renal disease^5^. Generally, kidney function after nephrectomy usually recovers up to 60-70% of baseline function through a compensatory hypertrophy mechanism^6^. However, it is still unclear why the degree of compensatory hypertrophy is variable between donors. One possible mechanism is the presence of subtle metabolic syndromes or preclinical renal diseases before donation^7^, which could potentially reduce the renal recovery capacity after donation.

JC polyomavirus (JCV) is a human polyomavirus that cause asymptomatic childhood infection and persists in various sites including the urinary tract^8^ and the central nervous system^9^. Nearly 80% of adults are seropositive for JCV^10^ and approximately 30% of the adult population shed JCV in urine at any time point^11^. With the exception of immunosuppression, factors that control the balance between latency and reactivation of polyomaviruses are unknown. Some studies unveiled a protective role of JCV against chronic kidney disease progression in selected populations^12,13,14,15^. Polyomavirus viruria is not routinely measured before kidney donation, and, as far as we know, the potential role of JCV viruria on the development of *de novo* cardiovascular risk factors, in the magnitude of kidney function decline, or in new onset kidney disease in LKD is unknown.

Few studies have investigated pre-donation factors that could affect the potential for recovery of kidney function after donation. Additionally, few studies evaluated the longitudinal performance of kidney function in LKD, which is preferable over a single point estimate.

Hence, we conducted a longitudinal retrospective study to investigate the role of JCV viruria and other pre-donation factors on the decline of kidney function after LKD.

## Methods

### Study design and population

In this single-center longitudinal retrospective study we enrolled 60 consecutive LKD (aged ≥18 years), followed in a Kidney Transplant Unit in Portugal, with at least one year of follow-up after donation.

From May 2014 to February 2020, all 60 donors collected serum and urine prior to donation to evaluate JCV serum and urinary viral load status. Urine and plasma JCV viral loads were measured by quantitative commercial real-time polymerase chain reaction (qPCR). Whenever the screening for polyomavirus viruria was positive, the persistence of viral shedding was confirmed through another qPCR measurement at least 3 months after the first determination to evaluate the consistency of viruria, avoid false positive results, and evaluate variations in viral load over time.

The study protocol complied with the Declaration of Helsinki and obtained full approval from the local clinical research ethics committee.

### Data collection and laboratory measurements

Demographic data of LKD (age, gender, and ethnicity) was recorded at baseline.

Before donation, GFR was measured through radioisotope renography and by classical creatinine measurement and was estimated through the Chronic Kidney Disease Epidemiology Collaboration (CKD-EPI) equation^16^. The 24-hour proteinuria was measured at baseline in morning sterile spot urine samples. LKD candidates with proteinuria ≥200 mg/24 hours were excluded as donors.

Before donation, blood pressure (BP) was evaluated in all LKD through 24-hour ambulatory blood pressure (ABPM) measurement. Hypertension was defined as mean systolic BP >130 mg, diastolic BP >80 mmHg, or a controlled BP with at least one antihypertensive drug. Donors with hypertension were accepted at our center if the following criteria were fulfilled: no end organ damage (left ventricular hypertrophy, proteinuria, abnormal fundoscopy) and average BP levels of 130/80 mmHg on ABPM under 2, or fewer antihypertensive drugs. After donation, BP was assessed by a physician in the follow-up visits as the mean value of 3 measurements.

LKD candidates with type 2 diabetes mellitus or glucose intolerance were excluded from donation.

Presence of cardiovascular risk factors (dyslipidemia and hypertension), urine protein/creatinine ratio, serum creatinine, and eGFR were collected at baseline and at post-donation follow up visits, which occurred at least once a year.

After donation, optimal compensatory hypertrophy was defined as an eGFR ≥60% of the pre-donation eGFR, while the group of suboptimal compensatory hypertrophy included all donors with less than 60% of the pre-donation eGFR. We compared both groups for the following outcomes: post-nephrectomy development of hypertension, type 2 diabetes mellitus, glucose intolerance, dyslipidemia, proteinuria, and magnitude of kidney function decline.

### JCV analysis

A commercial real-time PCR technique (JCV ELITe MGB Kit) was used for JCV viral load determination. Two amplification reactions were performed starting from extracted DNA. A specific primer for the Large T antigen region of the JCV gene and a specific primer for an artificial sequence of DNA (internal control) were used. The JCV specific probe with ELITe MGB^®^ technology, labelled with FAM fluorophore, is activated when it hybridizes with the specific product of JCV amplification reaction. Viral load is obtained through a calibration curve.

### Statistical analysis

Categorical variables were described as absolute or relative frequencies. Continuous variables were described as mean ± standard deviation (SD) for normally distributed variables and median values and interquartile range for non-normally distributed variables. Proportions were compared using chi-squared test. Differences between clinical data were assessed by Student’s t test for unpaired samples for normal variables and Wilcoxon test for continuous data with non-normal distribution. A P value of <0.05 was considered statistically significant. All statistical tests were performed using the Statistical Package for the Social Sciences (SPSS) 25.0 software (SPSS, Inc, Chicago, IL, USA).

## Results

A total of 60 LKD with a mean age of 50.9±12.0 years were enrolled in this study and followed-up for 3.2±1.6 years after donation.

Clinical and demographical data at baseline are detailed in Table 1. All donors had 24-hour proteinuria <200 mg before donation. The overall prevalence of JCV viruria before donation was 40.0% (n=24) and JCV viremia was absent in all LKD. JCV viruria was re-evaluated before donation in all 24 LKD. JCV viruria was consistent in both evaluations in all patients.

**Table 1:** Clinical and demographic data of LKD at baseline

Characteristics	All donors (n=60)	JCV viruric donors (n=24)	JCV non-viruric donors (n=36)	p value
Age - mean ± SD, years	50.9 ± 12.0	53.7 ± 13.1	49.1 ± 11.1	0.261
Male gender - n (%)	17 (28.3)	10 (41.7)	7 (19.4)	0.082
Caucasian - n (%)	57 (95)	24 (100)	33 (91.7)	0.268
Hypertension - n (%)	11 (18.3)	6 (25.0)	5 (13.9)	0.321
Serum creatinine - mean ± SD, years	0.8 ± 0.1	0.8 ± 0.1	0.8 ± 0.1	0.094
Smoke habits - n (%)	10 (16.7)	3 (12.5)	7 (19.4)	0.725
Dyslipidemia - n (%)	17 (28.3)	7 (29.2)	10 (27.8)	0.794
GFR (radioisotope renography) - mean ± SD, mL/min	93.5 ± 18.1	94.2 ± 20.2	93.1 ± 16.9	0.381
eGFR (CKD-EPI) - mean ± SD, mL/min	96.1 ± 16.7	91.8 ± 15.1	98.9 ± 17.3	0.595

LKD: living kidney donors; SD: standard deviation; GFR: glomerular filtration rate; eGFR: estimated glomerular filtration rate.

After donation, the average length of hospital stay was 7.3±1.7 days. At discharge, the mean eGFR was 62.1±14.9% of baseline eGFR.

### Comparison of baseline characteristics between JCV viruric and non-viruric LKD

JCV viruria was not associated with age (p=0.261), baseline hypertension (p=0.321), measured GFR (p=0.38), or any baseline clinical or demographical parameter (Table 1).

### Clinical outcomes after donation

Clinical outcomes after donation are described in Table 2. *De novo* hypertension was diagnosed in 5 patients (8.3%). Only one patient developed overt proteinuria (>200 mg/g) after donation. Dyslipidemia was diagnosed in 9 (15%) patients and glucose intolerance or type 2 diabetes mellitus in 8 (13.3%) patients.

**Table 2:** Clinical outcomes after donation

	Pre-donation (n=60)	1^st^ year after donation (n=56)	2^nd^ year after donation (n=45)	3^rd^ year after donation (n=36)	4^th^ year after donation (n=23)
eGFR (CKD-EPI) - mean ± SD, mL/min	96.1 ± 16.7	63.3 ± 14.7	63.1 ± 13.7	64.3 ± 14.3	64.9 ± 15.1
Recovery of renal function after donation - mean ± SD, %		66.1 ± 10.6	65.3 ± 11.4	66.9 ± 10.2	67.9 ± 13.2
Proteinuria >200 mg/g - n (%) (cumulative)	0 (0)	0 (0)	1 (1.7)	1 (1.7)	1 (1.7)
De novo Hypertension - n (%) (cumulative)		3 (5,4)	4 (6,7)	5 (8,3)	5 (8,3)

eGFR: estimated glomerular filtration rate; SD: standard deviation.

### Association of hypertension, gender and age with magnitude of decline of kidney function after LKD

No association was found between age, gender, and baseline hypertension with post-donation eGFR less than 60% of the pre-donation baseline function at 1^st^, 2^nd^, 3^rd^, and 4^th^ year after donation (Table 3).

**Table 3:** Associations between age, gender, and baseline hypertension with post-donation EGFR 1, 2, 3, and 4 years after donation

	post donation eGFR <60% of the pre donation baseline function	post donation eGFR >60% of the pre donation baseline function	p value
Age mean ± SD, years			
1^st^ year after donation	50.6±12.1	51.7±12.6	0.779
2^nd^ year after donation	52.4±14.5	50.3±11.8	0.600
3^rd^ year after donation	53.7±11.6	51.7±12.6	0.687
4^th^ year after donation	51.2±14.4	54.1±12.1	0.620
Male gender - n (%)			
1^st^ year after donation	5 (31.3)	11 (68.8)	1.000
2^nd^ year after donation	4 (25.0)	12 (75.0)	0.738
3^rd^ year after donation	4 (33.3)	8 (66.7)	0.443
4^th^ year after donation	3 (30.0)	7 (70.0)	1.000
Baseline hypertension - n (%)			
1^st^ year after donation	4 (36.4)	7 (63.6)	0.712
2^nd^ year after donation	3 (33.3)	6 (66.7)	1.000
3^rd^ year after donation	2 (28.6)	5 (71.4)	1.000
4^th^ year after donation	1 (33.3)	2 (66.7)	1.000

SD: standard deviation.

### Association of JCV viruria with development of hypertension and magnitude of decline of kidney function after LKD

**Figure 1:**
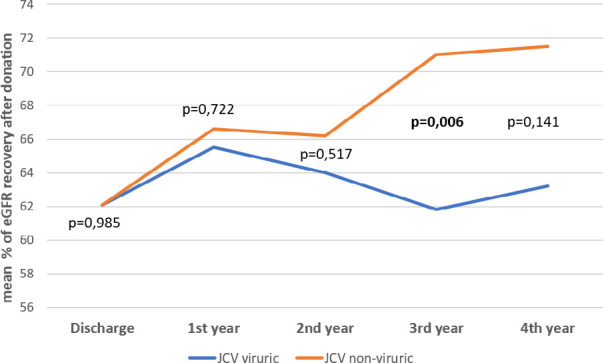
Comparison of mean % eGFR recovery after donation between JCV viruric and non-viruric donors.

No difference was found in mean eGFR recovery at discharge and at 1 and 2 years after donation between JCV viruric and non-viruric donors (Figure 1). Nevertheless, mean eGFR recovery 3 years after donation was lower in JCV viruric donors compared with non-viruric donors (61.8 vs 71.0%, p=0.006). At the 4^th^ year after donation, eGFR recovery was also lower in JCV viruric donors (63.2 vs 71.5%, p=0.141), although this difference did not reach statistical significance.

eGFR: estimated glomerular filtration rate.

JCV viruria was not associated with *de novo* hypertension after LKD (40 vs 60%, p=0.787).

## Discussion

In the present study we evaluated the role of JCV viruria on the magnitude of decline of kidney function after LKD for at least one year after donation. Additionally, we assessed the association of other pre-donation factors with measured renal function recovery after donation.

The risk of kidney dysfunction after LKD is a major concern, and therefore, it is crucial to optimize donor selection to reach the best outcomes after donation^17^. However, this risk is difficult to define, as GFR loss is physiologically observed with age. Therefore, the main question is whether kidney donation accelerates the decline in physiological GFR loss in addition to nephrectomy-related reduction in kidney mass and function, which is very difficult to demonstrate. In the present study, neither age, gender, or baseline hypertension were associated with achieving a post-donation eGFR less than 60% of the pre-donation baseline function 1, 2, 3, and 4 years after donation. Denic et al. investigated the relationships among kidney risk factors and revealed that mild hypertension and ageing were risk factors for underlying abnormalities such as nephrosclerosis and nephron hypertrophy in donors^18^. However, van Londen et al. showed that the GFR slope was not associated with hypertension, which could be explained by the practice of accepting only low-risk hypertensive donor candidates^19^.

Okumura et al. followed 133 LKD for one year and concluded that age, sex, and hypertension were significant preoperative predictors for patients who lost >40% of their eGFR 1 year after LKD^20^. Furthermore, Young et al. conducted a meta-analysis of 3 studies that assessed the post-donation change in eGFR in 23 older and 541 younger donors^21^. The meta-analysis found that older donors had a lesser decline in eGFR compared to younger donors (-6.38 mL/min; 95%CI:-2.56 to-10.21). Therefore, it was shown that older donors do not have a long-term deterioration of renal function, which is in line with our results.

Although we did not find an association between gender and renal function after donation, several studies revealed that the male gender is an independent risk factor for deterioration of renal function after donation^22,23^. The small number of patients with baseline hypertension (n=11) and male gender (n=17) could contribute to the lack of statistical association.

*De novo* hypertension was diagnosed in 5 patients (8.3%) after donation. The literature regarding the incidence and prevalence of hypertension following LKD is inconsistent in study design and outcomes^2^. Moreover, the prevalence of hypertension also increases with age in the general population. It is still unclear whether or not there is a significant risk of hypertension in donors compared to the general population. Nevertheless, hypertension is a heterogeneous phenomenon, affecting donors in different degrees and may be influenced by several factors^24^.

Londen et al. found that only 2% of donors developed proteinuria >0.5 g/day^19^ five years post donation. Similarly, in our study, only one patient developed overt proteinuria after donation.

After donation, dyslipidemia was diagnosed in 9 (15%) patients and glucose intolerance or type 2 diabetes mellitus in 8 (13.3%) patients. Data regarding the development of dyslipidemia after LKD are lacking. However, according to Holscher et al., the development of diabetes after LKD is a rare phenomenon^25^. The authors followed 41,260 LKD and showed that at 6 months, 1 year, and 2 years after donation, there were 2, 6, and 15 cases of diabetes per 10 000 donors, respectively. As our sample was small, it was impossible to achieve a definite conclusion.

JCV appears to establish a benign latent infection in reno-urinary epithelium with periodic reactivation and viral excretion in urine^11^. Nevertheless, asymptomatic urinary shedding of JCV has shown conflicting results. In a Brazilian study^26^, the prevalence of JCV viruria in a healthy control group was 20.1%. Moreover, Rodrigues et al^27^. noted a 23.9% urinary excretion of JCV in the general population in Portugal. However, in a group of 120 African American non-nephropathic individuals^13^, prevalence of JCV varied between 40% for APOL1 the renal-risk genotype group and 48.8% for APOL1 the non-risk genotype group. Our results are in line with the former study, considering the higher incidence of JCV viruria (40%).

We aimed to confirm the persistence of virus shedding before donation. JCV viruria was consistent between the 2 measurements in all 24 JCV viruric donors. Therefore, an isolated positive sample is enough to define the state of JCV urinary carrier.

Some recent reports have described a potential protective association between JCV viruria and kidney disease. Divers at al.^12^ tested whether infection by JCV and BKV modulated the association between APOL1 and development of nephropathy. They found that the presence of JCV viruria in patients with increased risk of APOL1-associated nephropathy was negatively associated with albuminuria and CKD (eGFR <60 mL/min/1.73 m^2^). The authors postulated that JCV may interact with APOL1 genotypes to modulate kidney disease risk^12^.

In a subsequent analysis^13^ of African American individuals with mild-severe CKD, JCV viruria was present in 45.8% of non-nephropathy controls and in 8.75% of CKD cases regardless of APOL1 renal-risk genotype status. The authors postulated that JCV had a robust CKD protective effect [OR (95% CI) 0.15 (0.06-0.42)]. These results were further extended to African Americans with diabetic kidney disease^14^ and to Black South Africans with hypertension-attributed CKD^15^.

Our study showed conflicting results. JCV viruria was not associated with any baseline parameter of LKD, including measured GFR or eGFR. This fact was probably due to the absence of patients with reduced renal function.

Post-donation renal function is approximately 60% of baseline function due to adaptive hyperfiltration and hypertrophy of the remaining kidney^6^. Unexpectedly, we found that mean eGFR recovery 3 years after donation was lower in JCV viruric donors compared with non-viruric donors (61.8 vs 71.0%, p = 0.006). Four years after donation, eGFR recovery was also lower in JCV viruric donors (63.2 vs 71.5%, p=0.141), although this difference did not reach statistical significance, probably due to the small number of patients in the analysis (n=23). The reason why JCV viruria associates with blunted kidney function recovery 3 years after donation remains undetermined.

After nephrectomy, the remaining kidney develops a functional adaptation through an increase in renal filtration of nephrons due to the increase renal plasma flow, which is accompanied by an increase in intraglomerular pressure^28^. Renal hyperfiltration and increase in intraglomerular pressure could eventually lead to a deleterious effect on kidney function with time. Furthermore, the reason why compensatory adaptation differs among donors is unclear. Previous studies postulated that preclinical renal diseases could affect renal recovery after donation^7^. We hypothesized that JCV could shift glomeruli into a hyperfiltration state before nephrectomy, modulating the magnitude of compensatory hypertrophy after kidney donation; thus, at mid-term, the potential of adaptive increase of GFR in these patients might be lower than in patients who did not shed JCV. Conversely, JCV might limit the ability of the remaining kidney to promote hyperfiltration, which might actually be a protective mechanism, as long-term hyperfiltration may be deleterious. Several approaches to protect against renal function deterioration target hyperfiltration, including renin-angiotensin-aldosterone system blockade^29^ or sodium-glucose cotransporter 2 (SGLT2) inhibition^30^. Longer follow-up is required to evaluate long-term effects on kidney function.

Our study has several limitations: it is a single-institution study with a small sample size. The follow-up of up to 4 years after donation is relatively short for evaluating the effects of baseline parameters on post-donation outcomes and on the development of *de novo* comorbidities. Furthermore, its retrospective nature does not allow to establish causality. Due to the low incidence of suboptimal compensatory adaptation in the remaining kidney, a more sophisticated statistical analysis, including multivariate analyses, was not possible. Also, we used estimated GFR to analyze the magnitude of kidney function decline after donation, which could overestimate decline^19^. However, the use of eGFR is common in most transplant centers^31^ and is in agreement with recent guidelines^32^.

A major strengths of our study is the longitudinal assessment of kidney function, unlike most studies that examine only a single time-point after donation. Furthermore, as far as we know, this is the first study to examine the role of pre donation JCV viruria in post-LKD outcomes. Longer follow-up is needed to determine whether JCV viruria ultimately leads to lower eGFR over time or if it is a protective factor for the remaining kidney.

In conclusion, although we are aware that these data cannot establish a cause-effect relationship, we believe that this is a hypothesis-generating article on a previously unknown association between JCV and blunted recovery of renal function after kidney donation. Therefore, these findings may draw attention to this possible association, which can be clarified in future larger prospective studies. In addition, two potential explanations for the role of JCV infection in glomerular hyperfiltration are suggested for future evaluation.
